# Study on the Freeze–Thaw Durability and Carbon Emission Reduction Benefits of Geopolymer EPS Concrete

**DOI:** 10.3390/ma19102023

**Published:** 2026-05-13

**Authors:** Xiaohong Jian, Haijie He, Ji Yuan, Haifei Lei, Shifang Wang, Yuhao Shang, Hanying Shou, Peixuan He, Zihang Ding, Ziyu Mao

**Affiliations:** 1 Zhejiang Reborn Kete Testing Co., Ltd., Hangzhou 311122, China; jianxiaohong1983@126.com (X.J.); lhf448613880@126.com (H.L.); 2 College of Civil and Architectural Engineering, Taizhou University, Taizhou 318000, China; he_haijie@zju.edu.cn (H.H.); mr_wsf@tzc.edu.cn (S.W.); messy6857@gmail.com (Y.S.); 2338420062@stu.tzc.edu.cn (H.S.); peixuanhe311@163.com (P.H.); zihangding002@163.com (Z.D.); ziyumao010@163.com (Z.M.)

**Keywords:** geopolymer EPS concrete, freeze–thaw resistance, microstructural testing, damage model, carbon emission assessment

## Abstract

In an effort to explore the influence mechanism of expanded polystyrene (EPS) foam particle content on the freeze–thaw resistance of geopolymer EPS concrete (GEPSC) and realize the synergistic optimization of freeze–thaw durability and low-carbon performance, systematic tests on the apparent morphology, mass loss rate, and relative dynamic elastic modulus (RDEM) of GEPSC with different EPS contents (30%, 35%, 40%, 45%, 50%, 55%) were conducted via freeze–thaw cycle tests. A parabolic damage model was established based on the theory of damage mechanics, and comparisons were made between GEPSC and conventional EPS concrete (EPSC) in terms of microstructure and carbon emission effect. Results indicate that the freeze–thaw resistance of GEPSC exhibits a nonlinear negative correlation with EPS content, which clarifies the applicable scope of GEPSC with different EPS dosages. The fitting correlation coefficient R^2^ of the established parabolic damage model is all higher than 0.98, which can accurately predict the evolution law of freeze–thaw damage of GEPSC. The interfacial transition zone of GEPSC is indistinct and the geopolymer matrix presents a denser structure. Compared with EPSC of the same density, the carbon emission of GEPSC is reduced by 45.3%, demonstrating that GEPSC integrates favorable freeze–thaw resistance with prominent environmental benefits. This study provides a scientific basis for the mixed proportion design and engineering application of low-carbon concrete materials in cold regions.

## 1. Introduction

Under the backdrop of the dual-carbon goals and the growing demand for energy conservation and carbon reduction in the construction industry, carbon emissions from cement production have been recognized as one of the major sources of anthropogenic carbon dioxide emissions globally [1,2,3]. Promoting the low carbonization of cementitious materials, resource utilization of solid wastes, and functional compounding has thus become a key development direction for concrete materials [4]. Expanded polystyrene (EPS) concrete, which uses EPS foam particles as lightweight aggregates, has attracted extensive attention and been widely applied in enclosure structures, lightweight wall panels, integrated thermal insulation systems, and building rehabilitation due to its advantages of light weight, thermal insulation, and ease of construction [5,6,7,8,9]. However, this material often faces an inherent trade-off: reduced density is accompanied by simultaneous degradation of strength and durability. Particularly, when serving in cold regions, it is more vulnerable to the challenges posed by freeze–thaw environments [10]. Therefore, while retaining the benefits of lightweight and thermal insulation, further reducing the carbon emissions of the cementitious system and addressing the durability shortcomings are core issues that urgently need to be resolved for the engineering popularization of EPS-based lightweight concrete.

Research on the freeze–thaw resistance of EPS concrete, both domestically and internationally, has mainly focused on two main lines: mix proportion parameter optimization and interfacial transition zone (ITZ) regulation. On the one hand, the compactness and pore structure of the cement paste are optimized by incorporating mineral admixtures such as silica fume and rice husk ash, thereby improving strength and durability. For instance, Sadrmomtazi et al. [11] investigated the effects of silica fume and rice husk ash on the mechanical and durability properties of EPS concrete with different strength grades, confirming that reactive admixtures can alleviate the performance degradation caused by EPS incorporation to a certain extent. Fine-scale filling and reinforcement methods, such as the use of microspheres adopted by He et al. [12], have also been applied to improve the pore structure and interfacial state of EPS concrete; their research showed that microspheres can enhance strength and increase the freeze–thaw resistance grade of EPS concrete. On the other hand, to address the weak interfacial bonding issue caused by the hydrophobicity and smooth surface of EPS particles, Yuan et al. [13] proposed a coating modification strategy. They conducted freeze–thaw durability studies based on the coating modification of EPS particles, pointing out that coating modification can significantly reduce ITZ defects and improve the freeze–thaw resistance grade. Meanwhile, they indicated that the EPS coating reinforcement technology can enhance interfacial hydrophilicity and compressive performance, providing a technical pathway for durability improvement. In addition, Dou et al. [14] found that fiber incorporation can inhibit crack development in EPS lightweight concrete, and hydrophobic modification can reduce the risks of water absorption and frost heave, offering another approach to enhance the freeze–thaw resistance of EPS concrete. In terms of the freeze–thaw mechanism, the freeze–thaw damage of EPS concrete is highly coupled with its pore structure, moisture migration, and interfacial defects. Factors such as EPS content, cementitious system compactness, ITZ quality, air-entraining system stability, and the synergistic effects of admixtures/mineral admixtures may alter the occurrence state of free water and the transmission path of frost heave pressure, thereby affecting degradation characteristics such as mass change, dynamic elastic modulus attenuation, and macro-scale spalling and cracking. It is worth noting that most existing studies have focused on cement-based EPS concrete, and the freeze–thaw evaluation indices and failure criteria for the EPS system may exhibit characteristics different from those of ordinary concrete. For example, the mass may increase in stages, and the dynamic elastic modulus may follow different change rules, leading to uncertainties in the evaluation of freeze–thaw durability and service life prediction [13].

Meanwhile, towards more low-carbon cementitious systems, geopolymers/alkali-activated materials have attracted considerable attention due to their ability to utilize solid wastes such as fly ash and slag and their low carbon footprint. However, systematic data support is still lacking regarding the interfacial structure, pore structure evolution, and freeze–thaw durability laws of geopolymers incorporated with EPS. Existing studies have mostly concentrated on the feasibility and basic properties of EPS application in lightweight geopolymers, such as lightweight performance, thermal properties, or other service scenarios [15], while research on the application scope and damage prediction under freeze–thaw environments in cold regions remains insufficient.

Based on the above background and research gaps, guided by the dual-carbon strategy, this paper proposes geopolymer EPS concrete (GEPSC) as a candidate material that integrates the properties of lightweight thermal insulation, solid waste utilization, and low-carbon footprint. This study systematically investigates the evolution laws and micro-mechanisms of freeze–thaw durability of GEPSC under different EPS volume contents, clarifying its engineering application scope. Simultaneously, the concept of damage mechanics is introduced to establish a freeze–thaw damage model, realizing quantitative characterization and prediction of performance degradation. Moreover, the evaluation of freeze–thaw durability is combined with the calculation of carbon emission per unit volume (CO_2_-e), aiming to achieve the synergistic optimization of freeze–thaw performance and low-carbon benefits. This research is expected to provide a scientific basis for the mix proportion design and engineering application of low-carbon lightweight concrete materials in cold regions.

## 2. Experimental Design

### 2.1. Raw Material

The sodium silicate solution used in this study was supplied by Henan Borun Foundry Materials Co., Ltd. (Zhengzhou, China), and its chemical composition and physical properties are listed in Table 1. Industrial-grade liquid sodium hydroxide with a concentration of 50%, produced by Shandong Feisheng Chemical Co., Ltd. (Dezhou, China), was used as the alkaline activator. The fine aggregate consisted of washed sea sand with an apparent density of 2630 kg/m^3^ and a fineness modulus of 2.64. Class II fly ash provided by Taizhou Tianda Environmental Protection Building Materials Co., Ltd. (Taizhou, China) was used, with a density of 2.09 g/cm^3^; its main chemical composition is presented in Table 2. The specific surface area of the fly ash ranged from 300 to 500 m^2^/kg, and its particle size distribution varied from 0.4 to 100 μm. Ground granulated blast-furnace slag (GGBS) of grade S95, with a density of 2.89 g/cm^3^, was employed as the mineral admixture, and its major chemical composition is also shown in Table 2. A polycarboxylate-based superplasticizer with a solid content of 30% was used to improve workability. The expanded polystyrene (EPS) beads had particle sizes ranging from 2 to 3 mm, the bulk density is 8.0 kg/m^3^.

### 2.2. Mix Proportion Design of GEPSC

Six groups of GEPSC specimens were designed in this study, while keeping the total amount of cementitious materials constant for all mixtures. Considering that the incorporation of EPS beads has varying effects on the mechanical performance and freeze–thaw resistance of concrete, six EPS content levels of 30%, 35%, 40%, 45%, 50%, and 55% were selected to investigate the evolution of concrete properties with increasing EPS content in geopolymer-based EPS concrete. During the mixing process of GEPSC, the water content was slightly adjusted according to the state of the slurry. The mix proportions of the six groups of geopolymer EPS concrete are shown in Table 3.

### 2.3. Specimen Preparation and Curing

**Figure 1:**
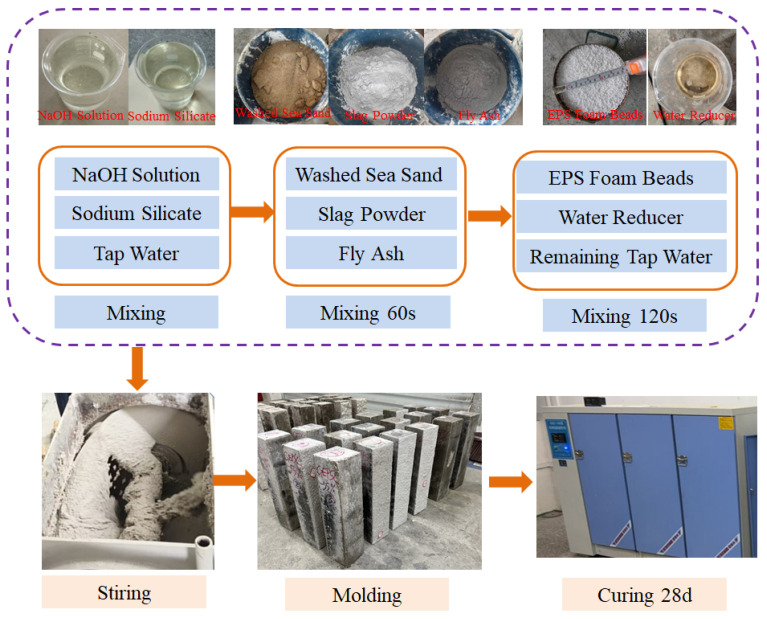
Main experimental procedure.

The alkaline activator used in this study was prepared by combining sodium hydroxide solution and sodium silicate solution. The designed sodium hydroxide solution, sodium silicate solution, and mixing water were blended to obtain the alkaline activating solution required for the experiments. During specimen preparation, washed sea sand, ground granulated blast-furnace slag, and fly ash were first placed into a mixer and dry-mixed for 60 s. Subsequently, the prepared alkaline activating solution was added and mixed until a uniform slurry was formed. EPS foam beads were then introduced, followed by the addition of the remaining mixing water. The main preparation procedure is illustrated in Figure 1.

Specimen preparation was carried out in accordance with the current national standard: Standard for Test Methods of Mechanical Properties of Concrete (GB/T 50081–2019) [16]. For each GEPSC mix proportion, three groups of cubic specimens (100 mm × 100 mm × 100 mm) and one group of prismatic specimens (100 mm × 100 mm × 400 mm) were prepared. Each group consisted of three specimens. One group of cubic specimens was used to determine the 28-day compressive strength, one group was used for dry density measurement, and one group was used for microstructural analysis. The prismatic specimens (100 mm × 100 mm × 400 mm) were used for freeze–thaw tests. After casting, all GEPSC specimens were stored in a natural curing environment. After 24 h, the specimens were demolded and transferred to a standard curing room, where they were cured until day 28.

### 2.4. Freeze–Thaw Test

In cold regions, freeze–thaw damage is one of the primary factors affecting the durability of concrete structures. During freeze–thaw cycles, the phase change in free water within the pores generates expansion pressure and hydraulic pressure inside the concrete, which can lead to progressive deterioration and even structural failure. According to the current national standard, Standard for Test Methods of Long-Term Performance and Durability of Ordinary Concrete (GB/T 50082–2024) [17], freeze–thaw resistance tests were conducted on GEPSC specimens with different mix proportions using the rapid freezing method.

In this study, the freezing temperature ranged from −20 to −18 °C with a freezing duration of 4 h, while the thawing temperature ranged from 18 to 20 °C with a thawing duration of 4 h. Clean water was used as the medium, and the specimens were fully submerged during the test. After every 15 freeze–thaw cycles, the mass of the specimens was measured using an electronic balance, and the relative dynamic elastic modulus was determined using a concrete dynamic modulus tester. The failure criteria for freeze–thaw damage of the specimens were as follows: (1) the mass loss rate exceeded 5%; and (2) the relative dynamic elastic modulus decreased by more than 60%. To investigate the freeze–thaw damage evolution of the specimens after reaching the failure criteria, the freeze–thaw test in this study was continued until the specimens fractured.

### 2.5. Microstructural Analysis

The microstructure of the specimens was characterized by scanning electron microscopy (SEM). A thin slice approximately 2 mm thick was taken from the central region of each GEPSC specimen for SEM observation. Prior to testing, the samples were dried and coated with a thin conductive metal layer to improve imaging quality.

SEM images were obtained at magnifications ranging from 100× to 50,000×. Detailed analyses were performed on the microstructure and hydration products of the GEPSC specimens, with particular attention paid to the interfacial contact between EPS foam beads and the geopolymer binder, as well as the gel morphology and pore structure within the matrix.

## 3. Test Results and Analysis

### 3.1. Density and Mechanical Properties

**Figure 2:**
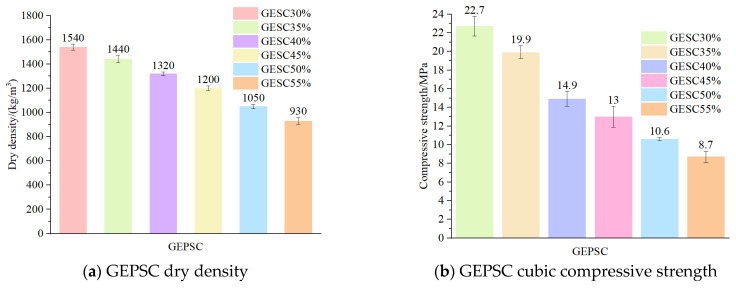
Dry density and cubic compressive strength of GEPSC.

It can be seen from Figure 2 that the dry density and cubic compressive strength of GEPSC show a monotonous decreasing trend with the increase in EPS content, and the changes in the two are consistent. The dry density gradually decreases from 1540 kg/m^3^ (GEPSC-30%) to 930 kg/m^3^ (GEPSC-55%), indicating that the overall lightweight effect of the system is significant with the increase in EPS particle content. Meanwhile, the cubic compressive strength decreases significantly from 22.7 MPa to 8.7 MPa, which shows that the strength is more sensitive to the EPS content. It can be concluded from the figure that GEPSC has a typical trade-off relationship between “lightweighting and strength loss”, and it is necessary to select an appropriate EPS content range between the target dry density and the engineering bearing requirements. To further clarify the influence of different binder systems on the mechanical performance of lightweight EPS concrete, GEPSC was compared with the EPSC reported in Ref. [12]. Considering that the mix design variables of the two material systems were not exactly the same, the comparison was conducted on the basis of the same density grade. At a dry density of 1100 kg/m^3^, the cube compressive strength of GEPSC-50% reached 10.6 MPa, which was significantly higher than the corresponding value of 7.09 MPa for EPSC in Ref. [12]. This indicates that, at the same lightweight level, the geopolymer binder system can form a matrix structure more favorable for strength development, thereby enabling GEPSC to exhibit superior mechanical performance.

### 3.2. Freeze–Thaw Damage Phenomenon

**Figure 3:**
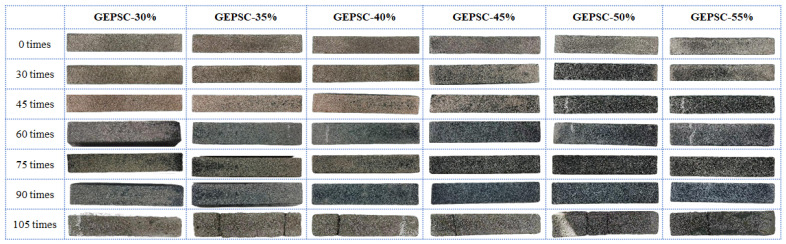
Freeze–Thaw Damage Morphology of Each Specimen.

Based on the observations of freeze–thaw cycle tests, the apparent morphology damage process of GEPSC series specimens is closely related to the volume content of EPS, showing obvious stage characteristics. Figure 3 illustrates the morphological damage of GEPSC caused by different freeze–thaw cycle times and EPS volume contents. Before 30 freeze–thaw cycles, the apparent morphology of all specimens remained basically intact without obvious damage. With the increase in freeze–thaw cycles, the damaged development of specimens with different EPS contents showed significant differentiation. After 45 freeze–thaw cycles, the apparent morphology of specimens GEPSC-30%~45% remained basically intact, while the geopolymer EPS foam particles on the surface of specimens GEPSC-50% and 55% began to be exposed. After 60 freeze–thaw cycles, slight damage occurred at the ends of specimens GEPSC-30% and 45%, with EPS foam particles falling off the surface; the apparent morphology of specimens GEPSC-35% and 40% remained basically intact. After 75 freeze–thaw cycles, the surface mortar of specimens GEPSC-35% and 40% began to spall, the geopolymer EPS foam particles on the surface started to be exposed and pitting corrosion formed on the surface, while no obvious changes were observed in other specimens compared with those after 60 cycles. After 90 freeze–thaw cycles, the surface mortar of low-content specimens (GEPSC-30%~40%) further spalled, the EPS foam particles wrapped in the internal geopolymer were obviously exposed, local pitting corrosion formed on the surface, accompanied by the generation of fine cracks; for high EPS content specimens (GEPSC-45%~55%), only local surface mortar spalling and a small amount of EPS particle exposure occurred, without obvious damage to the mortar on the side and bottom, and no crack propagation or structural damage was observed. After 105 freeze–thaw cycles, all six groups of specimens fractured with severe end damage. This process can be attributed to the repeated freezing and thawing of water in pores and incipient microcracks, which leads to the accumulation of internal damage and the complete failure of GEPSC.

### 3.3. Freeze–Thaw Resistance Characteristic

Freeze–thaw cycles tend to cause concrete to lose its excellent service performance and appearance integrity due to its inability to resist the action of environmental media for a long time. This section discusses the macroscopic changes in GEPSC during freeze–thaw cycles with respect to different volume contents of EPS foam particles.

#### 3.3.1. Mass Loss Rate

**Figure 4:**
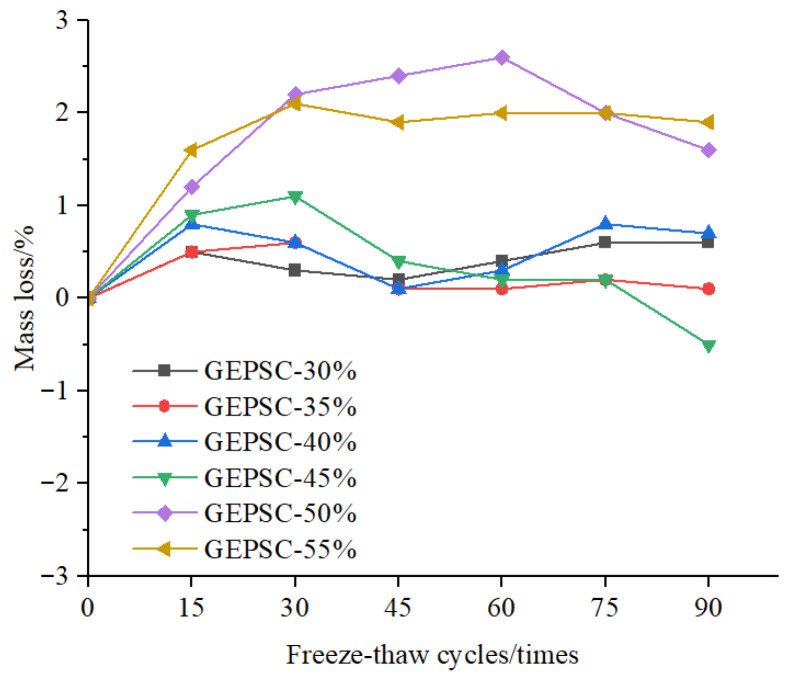
Mass change of GEPSC during freeze–thaw cycles.

The mass loss rate is an important indicator used to evaluate freeze–thaw damage in concrete. A positive value of the mass loss rate indicates mass loss, whereas a negative value indicates mass gain, meaning that the mass of absorbed water exceeds the mass lost due to surface spalling. The mass loss rates of six groups of GEPSC specimens with different volume contents of EPS foam particles during freeze–thaw cycles are shown in Figure 4. It can be seen from Figure 4 that during 0–30 freeze–thaw cycles, the mass loss rates of the high EPS foam particle content groups (GEPSC-50% and GEPSC-55%) increased significantly with the number of cycles, while the mass loss rates of the low EPS content groups (GEPSC-30%, GEPSC-35%, GEPSC-40%) and GEPSC-45% remained basically in the range of 0–1%, with a small amplitude of mass change. The reason is that after 0–30 freeze–thaw cycles, there are many pores between the internal EPS foam particles and the geopolymer matrix of the specimens in the high-content groups. Free water penetrates into the interior of the specimens along the pores, and no obvious damage is generated inside the specimens at this time, thus showing a trend of mass gain. In contrast, the matrix structure of the low-content groups is relatively dense with fewer pores, leading to limited water infiltration and thus slight mass changes. During 30–60 freeze–thaw cycles, the mass loss rates of GEPSC-50% and GEPSC-55% remained at a high level, while those of the other groups still kept fluctuating slightly. This is because the interface bonding between the matrix and EPS particles of the low-content groups is relatively tight, the pore expansion degree under freeze–thaw action is limited, and the water infiltration and loss are in a relatively balanced state, so the mass loss rates did not change significantly. During 60–90 freeze–thaw cycles, the mass loss rates of GEPSC-50% and GEPSC-55% began to decline, the mass loss rate of GEPSC-45% turned negative (with slight mass loss), while the mass loss rates of GEPSC-30%, GEPSC-35% and GEPSC-40% remained basically stable near zero. The possible reason is that for the high-content groups, as the number of freeze–thaw cycles increases, the interface frost heave damage between EPS foam particles and the matrix intensifies, resulting in the spalling of surface mortar and the shedding of EPS particles of the specimens, which causes mass loss and thus the decline of mass loss rates.

Overall, with the increase in the content of EPS foam particles, the mass loss of GEPSC basically increases and its freeze–thaw resistance gradually weakens. The mass loss rate of each specimen during the entire freeze–thaw cycle is ≤5%. Among them, the overall mass loss of specimens GEPSC-50% and GEPSC-55% is relatively large, indicating that the increase in EPS foam particle content leads to the weakening of freeze–thaw resistance.

#### 3.3.2. Dynamic Elastic Modulus Loss Rate

**Figure 5:**
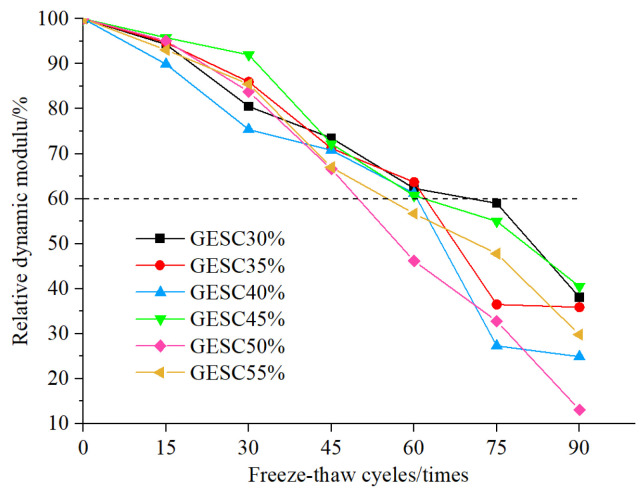
Dynamic elastic modulus change in specimens during freeze–thaw cycles.

The curves of relative dynamic elastic modulus changes in six groups of GEPSC specimens with different EPS foam particle contents after freeze–thaw cycles are shown in Figure 5. It can be seen from Figure 5 that during 0–30 freeze–thaw cycles, the relative dynamic elastic modulus of all GEPSC specimens showed a gentle downward trend but still remained at a high level. After 30 cycles, the relative dynamic elastic modulus was still maintained above 75%. During 30–60 freeze–thaw cycles, the decline rates of relative dynamic elastic modulus of specimens with different contents began to diverge. The downward trends of GEPSC-30% and GEPSC-35% were relatively gentle; after 60 cycles, the relative dynamic elastic modulus of GEPSC-50% and GEPSC-55% dropped below 60%, while that of the other specimens was still above 60%. In this stage, the interface damage of specimens GEPSC-30%~GEPSC-45% was still controllable and the surface remained basically intact, whereas the interface frost heave damage of specimens GEPSC-50% and GEPSC-55% accumulated, and a small number of geopolymer-EPS foam particles on the surface were exposed, leading to an accelerated decline in relative dynamic elastic modulus. During 60–90 freeze–thaw cycles, the performance differences among specimens with different contents further widened and the decline rate accelerated, indicating a further degradation of the mechanical properties of the specimens; after 75 cycles, the relative dynamic elastic modulus of all specimens dropped below 60%. At this time, the relative dynamic elastic modulus of specimen GEPSC-30% decreased the least, dropping to approximately 60%. This is because the interface bonding force of specimens with low EPS content can inhibit the propagation of frost heave cracks and maintain a certain structural integrity. In contrast, the interface damage of specimens with high EPS content has developed into macroscopic cracks, accompanied by EPS particle shedding and mortar spalling, resulting in severe internal structural damage and thus a significant reduction in relative dynamic elastic modulus.

Overall, with the increase in the number of freeze–thaw cycles, the decline rate of the relative dynamic elastic modulus of GEPSC gradually accelerates and its resistance to freeze–thaw damage gradually weakens. When the relative dynamic elastic modulus of all specimens drops below 60%, the GEPSC with 30% EPS content maintains the optimal relative dynamic elastic modulus and structural integrity throughout the freeze–thaw process, indicating that the specimens at this content level possess the strongest freeze–thaw resistance.

#### 3.3.3. Durability Index

The freeze–thaw durability index of GEPSC is determined in accordance with the current national standard: (GB/T 50082-2024) [17]. Combined with the judgment based on test phenomena, the frost-resistance grade is determined by the maximum number of freeze–thaw cycles when the relative dynamic elastic modulus is not less than 60% or the mass loss rate does not exceed 5%. In this test, the use of relative dynamic elastic modulus to determine the frost-resistance index is relatively more accurate.

**Figure 6:**
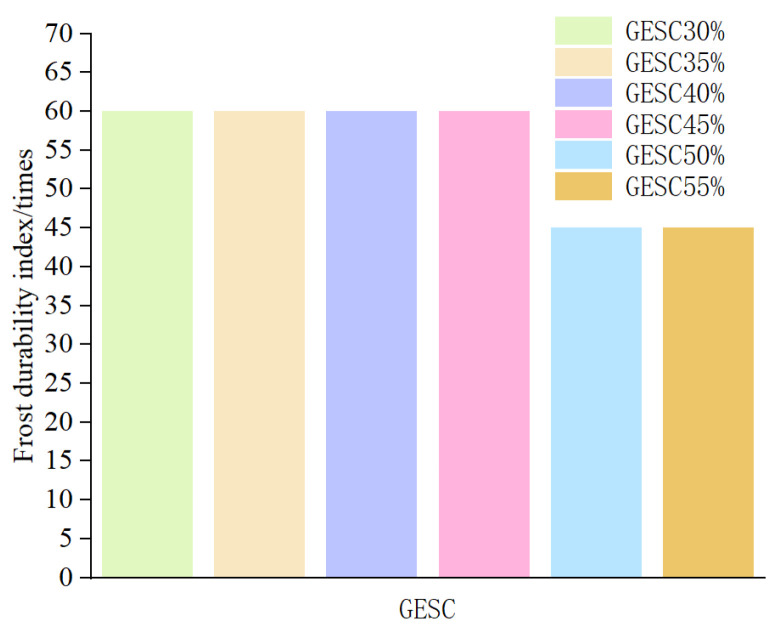
Frost-resistance index of GEPSC.

The distribution of freeze–thaw durability indexes of six groups of GEPSC with different volume contents of EPS foam particles is shown in Figure 6. The frost-resistance indexes of GEPSC-30%, GEPSC-35%, GEPSC-40% and GEPSC-45% specimens are consistent, all reaching 60 freeze–thaw cycles. By contrast, the durability indexes of GEPSC-50% and GEPSC-55% specimens are significantly lower, with the frost-resistance index at 45 freeze–thaw cycles. The freeze–thaw resistance of GEPSC is superior to that of EPSC at the same density grade, and its frost-resistance index is increased by 3–4 times [12].

According to the provisions of the current industry standard (JG/T 350-2011) [18], the frost resistance requirements of lightweight concrete slabs under different service conditions are shown in Table 4. Combined with Figure 6 and Table 4, it can be seen that the freeze–thaw cycle number of GEPSC with EPS foam content below 45% is more than 60, which meets all the service environments specified in Table 4. When the EPS foam particle content is higher than 50%, the frost resistance index of GEPSC is 45 freeze–thaw cycles, which is lower than F50. In this case, GEPSC can be applied in all regions except severe cold areas.

### 3.4. Microscopic Characterization of GEPSC

**Figure 7:**
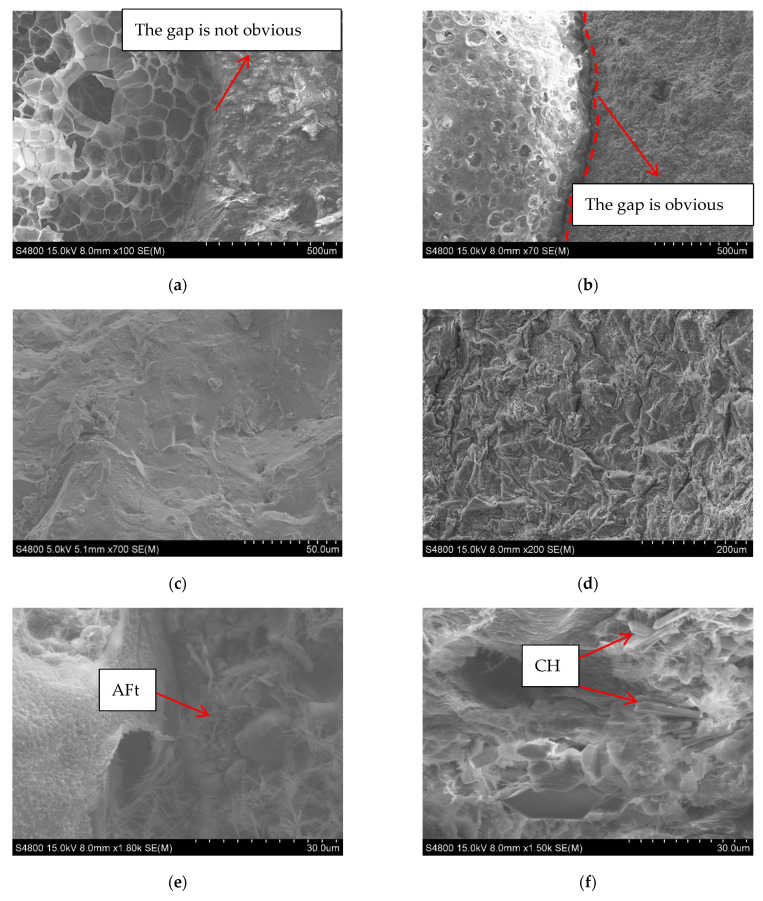
Microstructures of GEPSC and EPSC. (**a**) Interfacial Transition Zone of GEPSC. (**b**) Interfacial Transition Zone of EPSC [12]. (**c**) GEPSC Matrix. (**d**) GEPSC Matrix. (**e**) EPSC Matrix. (**f**) EPSC Matrix [12].

Figure 7 shows the SEM comparison between GEPSC and EPSC. It can be seen from Figure 7 that there are significant differences in the morphology of the cementitious matrix and interfacial characteristics of the two systems. The geopolymer matrix exhibits an overall denser and more uniform microstructure with fewer pores and no obvious penetrating cracks. In the interfacial transition zone (ITZ) between geopolymer and EPS (Figure 7a), the matrix shows good wrapping and filling effects on EPS particles. There are almost no obvious pores and microcracks near the interface, and even a phenomenon of “indistinct interface identification” appears in some areas. This may be related to the gel network dominated by sodium-aluminum-silicon-hydrate gel (N-A-S-H) in the geopolymer system. Unlike the traditional cement system, it does not necessarily form a “weak ITZ” around EPS particles. The interfacial area is more continuous with fewer pores and microcracks, reflecting better compatibility and structural integrity [19]. In contrast, a relatively distinct interfacial transition zone (ITZ) exists between the EPS particles and the matrix in cement-based EPS concrete (EPSC) (Figure 7b). The interface surrounding the EPS is relatively smooth, but the hydration products are relatively insufficient, and pores tend to accumulate at the interface, rendering the ITZ relatively weak. The cementitious phase of ordinary cement-based EPS concrete is mainly composed of calcium silicate hydrate (C-S-H) gel, calcium hydroxide (CH), and ettringite (AFt) (Figure 7e). Among these components, C-S-H mostly presents a layered or fibrous amorphous morphology and serves as the key component for bearing strength. In contrast, CH exists in a crystalline state, often in the form of hexagonal flakes (Figure 7f). Its presence tends to cause insufficient local compactness, which in turn results in the overall porosity of the interface and the width of the transition zone between the cement matrix and EPS being larger than those of the geopolymer system. At the same time, the geopolymer matrix (Figure 7c,d) is dominated by amorphous gel products. The amorphous gel generated by the reaction can effectively fill pores and refine the pore size distribution, thereby forming a denser pore structure with a more uniform pore size. This is also an important microscopic reason why geopolymer EPS concrete exhibits superior durability and impermeability.

## 4. Freeze–Thaw Damage Model

The damage degree of GEPSC for each group in this experiment is presented in Table 5. In the assessment of freeze–thaw performance improvement for GEPSC, the damage variable, as a core concept in the field of damage mechanics, is primarily used to quantitatively describe the development state of internal defects in the material. As freeze–thaw cycles progress, the initially existing micro-cracks and pores within GEPSC gradually expand and interconnect, leading to progressive damage and deterioration of the material’s macroscopic mechanical properties, which can ultimately cause irreversible freeze–thaw failure of the GEPSC specimens. To more accurately quantify and predict this freeze–thaw damage degree, this paper proposes a simplified parabolic damage model, which better conforms to the damage evolution law of GEPSC under freeze–thaw cycling and can more accurately reflect the damage development state during the material’s freeze–thaw process compared to traditional models. The theoretical damage model for GEPSC can be referenced from Equation (1) [20]:

(1) $$D=1-\frac{E_{n}}{E_{0}}=\frac{1}{2}e_{g}t^{2}$$

where D is the damage degree of GEPSC after n freeze–thaw cycles; n represents the number of freeze–thaw cycles; E_0_ and En are the dynamic elastic moduli of the CGC before and after n freeze–thaw cycles, respectively; e_g_ is the acceleration of RDEM damage; and t is the duration.

By introducing the number of freeze–thaw cycles n, the parabolic model of Equation (1) is adjusted and optimized to:

(2) $$D=1-\frac{E_{n}}{E_{0}}=in^{2}+jn+k$$

where i, j, k all represents fitting parameters.

(3) $$\frac{dD}{dn}=2in+j$$

(4) $$D{|}_{n=0}=k,\frac{dD}{dn}{|}_{n=0}=j$$

**Figure 8:**
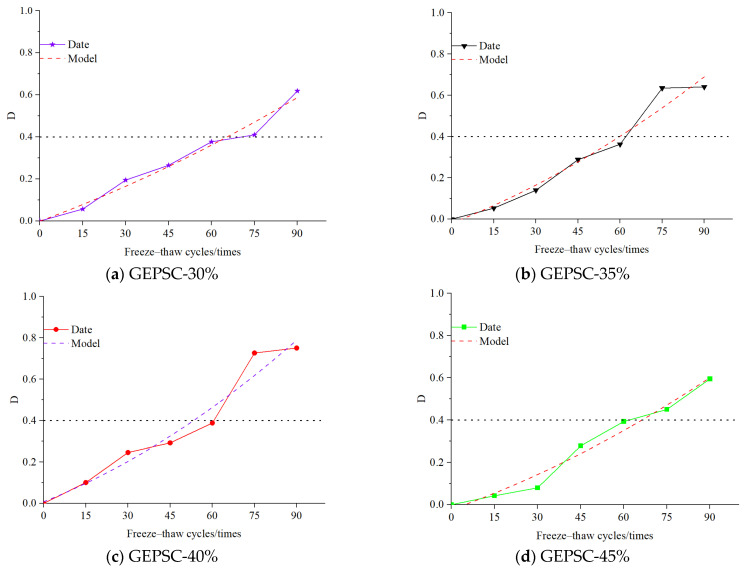

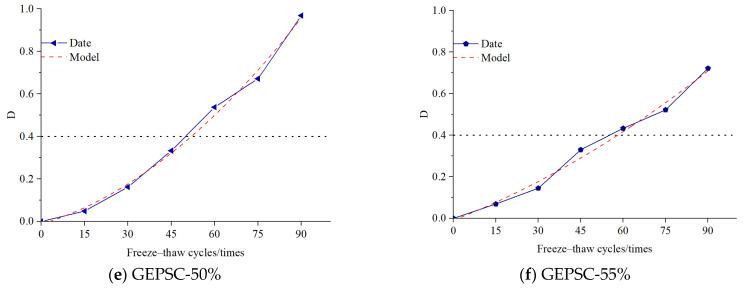
Fitting of damage degree under different bead contents.

Based on the parabolic damage model established by Equations (2)–(4), and using the relative dynamic elastic modulus (RDEM) test data of geopolymer EPS concrete (GEPSC) with different EPS bead contents, the freeze–thaw damage degree of each GEPSC specimen group was fitted. The specific damage model fitting parameters are shown in Table 6, which lists the fitting parameters i, j, k, and the correlation coefficient R^2^ for each group. The damage model fitting results are shown in Figure 8.

From the fitting results, the experimental damage degree data for each GEPSC group in Figure 8 shows good agreement with the parabolic model fitting curves. The fitting curves for GEPSC-50% and GEPSC-55% almost overlap with the experimental data, while the fitting curves for GEPSC-30%, GEPSC-40%, and GEPSC-45% show a good fit to the trend of the experimental data, intuitively demonstrating the model’s suitability for GEPSC freeze–thaw damage. Combined with Table 6, the correlation coefficients R^2^ for each group range from 0.9562 to 0.99482. Specifically, R^2^ for GEPSC-50% reaches 0.99482, and for GEPSC-55% it is 0.98937. The high correlation coefficients for most groups effectively prove that this parabolic damage model has high accuracy and reliability in predicting the freeze–thaw damage of GEPSC.

As shown in Table 5, the damage degree of GEPSC in each group generally increased with the number of freeze–thaw cycles., especially after 90 cycles, where the damage degree rises significantly. Furthermore, GEPSC with higher EPS bead contents (e.g., 50% and 55%) exhibits greater damage degrees compared to those with lower contents (e.g., 30% and 35%), indicating that higher bead content leads to more severe damage under freeze–thaw conditions.

## 5. Carbon Emission Assessment of GEPSC

In the sustainability assessment system for building materials, accurate measurement of their environmental impact is crucial. For GEPSC, the total carbon dioxide emissions per unit volume of concrete (CO_2_-e) are used as a key indicator to evaluate its environmental performance. Carbon dioxide equivalent (CO_2_-e) allows for the comprehensive consideration of the potential impact of various greenhouse gases on global warming by converting them into a unified equivalent, thereby facilitating the quantitative assessment of the environmental friendliness of GEPSC. Table 7 presents the carbon emission factors for each material. The sodium hydroxide used in this study has a concentration of 50%, and calculations are based on this concentration. The designed mix uses 22 kg/m^3^ of pure sodium hydroxide, with a carbon emission factor of 0.86 for pure sodium hydroxide. The carbon emissions reveal the CO_2_ emissions corresponding to the production process of each material and energy consumption. CO_2_-e is calculated according to Equation (5) [21,22,23]:

(5) $$CO_{2}-e=\sum_{i=1}^{n}\left(Q_{i}\times CO_{2\left(i\right)}\right)$$

where refers to the CO_2_ emissions generated per cubic meter of GEPSC (concrete waste), Q~i~ represents the mass of each material per cubic meter of GEPSC, and represents the CO_2_ emission factor of each material.

**Figure 9:**
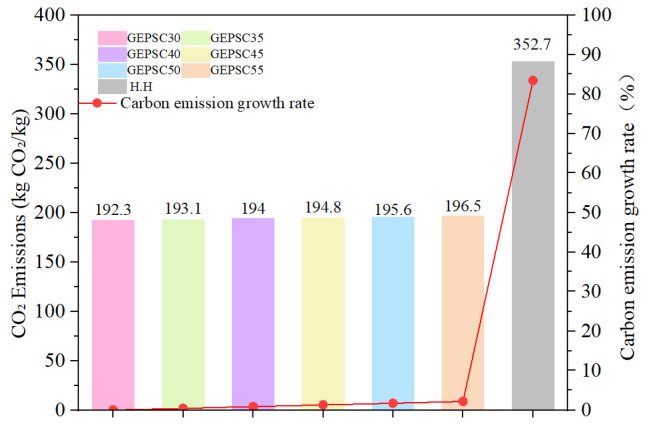
Carbon emissions of GEPSC and EPSC.

Figure 9 and Table 7 present the analysis of CO_2_ emissions per unit volume. As shown in Figure 9, the carbon emissions of GEPSC increase with higher EPS bead content, primarily due to the relatively high carbon emission factor of EPS beads. Additionally, the sample labeled H.H represents EPSC with a density grade of 1100 kg/m^3^. In this study, GEPSC-35% also has a density of 1100 kg/m^3^, and its carbon emissions are 45.3% lower than those of the EPSC. This indicates that GEPSC has a clear advantage in reducing CO_2_ emissions. GEPSC not only exhibits significant low-carbon advantages but also retains the lightweight and thermal insulation characteristics of EPS, offering prominent benefits in energy saving and emission reduction.

## 6. Conclusions

Based on the investigation of the freeze–thaw resistance and carbon emissions of GEPSC with different EPS bead contents, the following conclusions can be drawn:

(1) The freeze–thaw resistance of GEPSC decreases with increasing EPS bead content. When the EPS bead content is lower than 50%, the freeze–thaw resistance index of GEPSC exceeds 60 cycles, indicating that GEPSC lightweight exterior wall panels are suitable for application in various environmental conditions. However, when the EPS bead content exceeds 50%, GEPSC is not recommended for use in severe cold regions.
(2) The interfacial transition zone between the geopolymer gel matrix and EPS beads in GEPSC is denser and exhibits improved bonding performance. This enhancement is mainly attributed to the formation of a continuous network of N–A–S–H amorphous gel generated during the geopolymerization reaction, which effectively coats the EPS bead surfaces and fills internal pores.
(3) The calculated damage degree obtained from the proposed freeze–thaw damage model for GEPSC is in good agreement with the experimental results. The fitted model accurately predicts the damage evolution characteristics of GEPSC under freeze–thaw cycles, indicating that the damage model can effectively describe the freeze–thaw deterioration behavior of GEPSC.
(4) Compared with conventional EPS concrete (EPSC), GEPSC exhibits superior environmental performance. At the same density level, the carbon emissions of GEPSC are reduced by 45.3% compared to EPSC, demonstrating that GEPSC combines favorable thermal insulation performance with low carbon emissions.

## Figures and Tables

**Table 1:** Chemical composition and physical properties of sodium silicate solution.

Material	SiO_2_/%	Na_2_O/%	Density/(g/cm^3^)	Baumé Degree/Bé	Modulus/M	Transparency
Sodium silicate	27.5–30.5	8.5–10.5	1.394–1.408	41–42	3.32	≥85

**Table 2:** Chemical composition and physical properties of fly ash and slag.

Chemical Composition (%)	Al_2_O_3_	SiO_2_	CaO	MgO	Fe_2_O_3_	Na_2_O	K_2_O	SO_3_	TiO_2_
Fly ash	42.35	34.48	4.62	0.60	9.88	0.48	1.42	2.15	2.51
Ground granulated blast-furnace slag	15.7	30.8	41.5	7.7	0.3	0.7	/	1.1	/

**Table 3:** Mix proportions of GEPSC.

Mix ID	Fly Ash, kg	Slag, kg	Sand, kg	Sodium Hydroxide, kg	Sodium Silicate, kg	Water, kg	Superplasticizer, kg	EPS Mass, kg	EPS Volume Content,%
GEPSC30	110	440	800	44	185	260.5	7	3.84	30%
GEPSC 35	110	440	700	44	185	261.2	7	4.48	35%
GEPSC 40	110	440	600	44	185	262.7	7	5.12	40%
GEPSC 45	110	440	500	44	185	263.2	7	5.76	45%
GEPSC 50	110	440	400	44	185	266.4	7	6.40	50%
GEPSC 55	110	440	300	44	185	267.6	7	7.04	55%

**Table 4:** Frost resistance of lightweight concrete slabs for external walls [18].

Service Environmental Conditions	Frost-Resistance Grade	Indicator/Index
Hot Summer and Warm Winter Zone	F15	No visible cracks shall appear and the surface shall remain unchanged
Hot Summer and Cold Winter Zone	F25	
Cold Zone	F35	
Severe Cold Zone	F50	

**Table 5:** Damage degree of GEPSC.

Freeze–Thaw	GEPSC-30%	GEPSC-35%	GEPSC-40%	GEPSC-45%	GEPSC-50%	GEPSC-55%
0	0	0	0	0	0	0
15	0.057	0.053	0.101	0.042	0.049	0.069
30	0.195	0.14	0.246	0.08	0.162	0.145
45	0.265	0.288	0.292	0.278	0.333	0.33
60	0.377	0.363	0.388	0.393	0.538	0.433
75	0.41	0.635	0.727	0.45	0.672	0.522
90	0.619	0.641	0.751	0.595	0.969	0.722

**Table 6:** Fitting parameters of the GEPSC damage model.

Group	GEPSC-30%	GEPSC-35%	GEPSC-40%	GEPSC-45%	GEPSC-50%	GEPSC-55%
i (×10^−6^ per cycle^2^)	0.168785	0.28783	0.362433	0.234921	0.747616	0.294907
j (×10^−4^ per cycle)	50.2	52.9	54.2	48.2	40.5	53.4
k	−0.000403764	−0.0194	0.00788	−0.02321	−0.01214	−0.00934
R^2^	0.97725	0.96491	0.9562	0.97385	0.99482	0.98937

**Table 7:** Carbon emission factors of raw materials and carbon emissions of concrete.

Group	Emission Factor (kgCO_2_/kg)	GEPSC-30	GEPSC-35	GEPSC-40	GEPSC-45	GEPSC-50	GEPSC-55	H.H [12]
Cement	0.855 [24]	-	-	-	-	-	-	316.35
FA	0.009 [24]	0.99	0.99	0.99	0.99	0.99	0.99	3.33
GGBS	0.0804 [25]	35.38	35.38	35. 38	35.38	35.38	35.38	0
Sand	0.03 [26]	24.00	21.00	18.00	15.00	12.00	9.00	0
NaOH	0.86 [27]	18.92	18.92	18.92	18.92	18.92	18.92	0
Sodium silicate	0.43 [27]	79.55	79.55	79.55	79.55	79.55	79.55	0
SP	1.48 [28]	10.36	10.36	10.36	10.36	10.36	10.36	5.18
Water	0.000168 [29]	0.04	0.04	0.04	0.04	0.04	0.04	0.04
EPS	6.0 [30]	23.04	26.88	30.72	34.56	38.40	42.24	27.84
CO_2_-e (kgCO_2_/m^3^)	-	192.28	193.12	193.96	194.8	195.64	196.48	352.74

## Data Availability

The original contributions presented in this study are included in the article. Further inquiries can be directed to the corresponding author.

## Nomenclature

EPS	Expanded polystyrene
EPSC	Expanded polystyrene concrete
GEPSC	Geopolymer EPS concrete
AFt	Calcium sulfoaluminate hydrate
GGBS	Ground granulated blast-furnace slag
RDEM	Relative Dynamic Elastic Modulus
CH	Calcium hydroxide
FA	Fly ash
SP	Superplasticizer
ITZ	Interfacial transition zone
